# A new genus of Coelotinae (Araneae, Agelenidae) from southern China

**DOI:** 10.3897/zookeys.541.6678

**Published:** 2015-12-01

**Authors:** Lu Chen, Shuqiang Li, Zhe Zhao

**Affiliations:** 1 College of Life Sciences, Hebei University, Baoding, Hebei 071002, China; 2 Institute of Zoology, Chinese Academy of Sciences, Beijing 100101, China

**Keywords:** Taxonomy, spider, coelotine, SE Asia, Guanxi, Yunnan

## Abstract

One new genus of the spider subfamily Coelotinae, *Flexicoelotes* **gen. n.**, with five new species is described from southern China: *Flexicoelotes huyunensis* **sp. n.** (female), *Flexicoelotes jiaohanyanensis* **sp. n.** (male and female), *Flexicoelotes jinlongyanensis* **sp. n.** (male and female), *Flexicoelotes pingzhaiensis* **sp. n.** (female), *Flexicoelotes xingwangensis* **sp. n.** (male and female).

## Introduction

Coelotine spiders are common in the northern hemisphere. So far, a total of 646 valid species belonging to 23 genera (Wang 2012, Kim and Ye 2013, Kim and Ye 2014, Seo 2014, Ye and Kim 2014, Chen et al. 2015, Jiang and Chen 2015) are known worldwide, and 19 genera are known in Asia. The genera *Alloclubionoides* Paik, 1992, *Hypocoelotes* Nishikawa, 2009, *Tegecoelotes* Ovtchinnikov, 1999 are distributed in Far East Russia and East Asia. The other 16 genera: *Bifidocoelotes* Wang, 2002, *Coelotes* Blackwall, 1841, *Draconarius* Ovtchinnikov, 1999, *Femoracoelotes* Wang, 2002, *Himalcoelotes* Wang, 2002, *Iwogumoa* Kishida, 1955, *Leptocoelotes*, Wang 2002, *Lineacoelotes* Xu, Li & Wang, 2008, *Longicoelotes* Wang, 2002, *Notiocoelotes* Wang, Xu & Li, 2008, *Orumcekia* Koçak & Kemal, 2008, *Pireneitega* Kishida, 1955, *Platocoelotes* Wang, 2002, *Robusticoelotes* Wang, 2002, *Spiricoelotes* Wang, 2002 and *Tonsilla* Wang & Yin, 1992, are distributed in southern China and adjacent regions (Japan, Laos and northern Vietnam).

Wang (2012) revised most of the coelotine spiders based on type material. Twenty-two new coelotine species were reported from China and adjacent regions after 2012. Among them, 6 were found in southern China (Chen et al. 2015, Jiang and Chen 2015), 9 were known from Korea (Kim and Ye 2013, Kim and Ye 2014, Seo 2014, Ye and Kim 2014), and 7 were known from Japan (Koumura 2013).

In this paper, we describe a new genus of Coelotine spiders, *Flexicoelotes* gen. n., and five new species. All species were collected from caves in Guangxi and Yunnan, China.

## Materials and methods

Specimens were examined with a Leica M205C stereomicroscope. Images were captured with an Olympus C7070 wide zoom digital camera (7.1 megapixels) mounted on an Olympus SZX12 dissecting microscope. Epigynes and male palps were examined after dissection from the spiders’ bodies. The epigyne was cleared by boiling it in a 10% KOH solution before taking photos of the vulva.

All measurements were obtained using a Leica M205C stereomicroscope and are given in millimeters. Leg measurements are given as: Total length (femur, patella + tibia, metatarsus, tarsus). Only structures (palp and legs) of the left side of the body are described and measured. The terminology used in the text and the figure legends follows Wang (2002). Abbreviations used in this paper and in the figure legends are: A = epigynal atrium; ALE = anterior lateral eye; AA = anterior apophysis; AME = anterior median eye; AME-ALE = distance between AME and ALE; AME-AME = distance between AME and AME; ALE-PLE = distance between ALE and PLE; C = conductor; CD = copulatory duct; CDA = dorsal conductor apophysis; CF = cymbial furrow; E = embolus; EB = embolic base; ET = epigynal teeth; FD = fertilization duct; H = epigynal hood; LTA = dorso-retrolateral tibial apophysis; MA = median apophysis; PA = patellar apophysis; PLE = posterior lateral eye; PME = posterior median eye; PME-PLE = distance between PME and PLE; PME-PME = distance between PME and PME; RTA = retrolateral tibial apophysis; S = spermatheca; SH = spermathecal head; SST = spermathecal stalk; ST = subtegulum; T = tegulum.

A partial fragment of the mitochondrial gene cytochrome oxidase subunit I (COI) was amplified and sequenced for *Flexicoelotes huyunensis* sp. n., *Flexicoelotes jiaohanyanensis* sp. n., *Flexicoelotes jinlongyanensis* sp. n., *Flexicoelotes pingzhaiensis* sp. n., and *Flexicoelotes xingwangensis* sp. n. following the protocol in Miller et al. (2009). Primers used in this study are: LCO1490 (5’-CWACAAAYCATARRGATATTGG-3’) (Folmer et al. 1994) and HCO2198zz (5’-TAAACTTCCAGGTGACCAAAAAATCA-3’) (this study). All sequences were blasted in GenBank. The accession numbers are provided in Table 1.

**Table 1.** Voucher specimen information.

Species	GenBank accession number	Sequence length	Collection localities
*Flexicoelotes huyunensis* sp. n.	KT727020	1194 bp	TanjiawanVillage, Malipo County, Wenshan Prefecture, Yunnan Province, China
*Flexicoelotes jiaohanyanensis* sp. n.	KT727021	1194 bp	Equan Village, Jingxi County, Baise City, Guangxi Zhuang Autonomous Region, China
*Flexicoelotes jinlongyanensis* sp. n.	KT727018	1194 bp	Yongning Village, Napo County, Baise City, Guangxi Zhuang Autonomous Region, China
*Flexicoelotes pingzhaiensis* sp. n.	KT727019	1194 bp	Pingzhai Village, Xichou County, Wenshan Prefecture, Yunnan Province, China
*Flexicoelotes xingwangensis* sp. n.	KT727017	1194 bp	Xingwang Village, Debao County, Baise City, Guangxi Zhuang Autonomous Region, China

All of the specimens (including molecular vouchers) are deposited in the Institute of Zoology, Chinese Academy of Sciences (IZCAS) in Beijing, China.

## Systematics

### Family Agelenidae C.L. Koch, 1837 Subfamily Coelotinae F.O.P.-Cambridge, 1893

#### Genus Flexicoelotes gen. n.

Taxon classificationAnimaliaAraneaeAgelenidae

http://zoobank.org/3F8CE486-FF6F-40B1-926B-9F605AC5A40D

##### Type species.

*Flexicoelotes jiaohanyanensis* sp. n.

##### Etymology.

The generic name is derived from the species’ similarity to *Coelotes* and the Latin adjective “flexus”, meaning “bent, curved”, referring to the shape of the conductor. The gender is masculine.

##### Diagnosis.

Males can be easily distinguished from other coelotines, except *Tonsilla* Wang & Yin, 1992 and *Lineacoelotes* Xu, Li & Wang, 2008, by the broad conductor, the spoon-like median apophysis, and the elongate cymbial furrow. They can be distinguished from *Tonsilla* by the bent conductor apex, rather than a lobed conductor, the presence of an anterior apophysis, and the broad cymbial furrow (Fig. 2A–C; Wang and Yin 1992: figs 3–5). They can be distinguished from *Lineacoelotes* by the broad, bent and less modified conductor, the presence of an anterior apophysis, and the thin, simple patellar apophysis (Fig. 2A–C; Xu et al. 2008: figs 13–15). Females can be easily distinguished from other coelotines, except *Tonsilla* and *Lineacoelotes*, by the long epigynal teeth and the absence of epigynal hoods. They can be distinguished from *Tonsilla* by the large and simple atrium, rather than a posteriorly extended anterior atrial margin, an atrium with the anterior part wider than the posterior part, epigynal teeth that are separated rather than near one another, the short and posteriorly located spermathecae, and the broad, long copulatory ducts (Fig. 3A–B; Wang and Yin 1992: figs 8–10). They can be distinguished from *Lineacoelotes* by the large atrium, the short, simple spermathecae, and the absence of a long, coiled spermathecal head (Fig. 3A–B; Xu et al. 2008: figs 11–12).

##### Description.

*Flexicoelotes* are small to medium-sized, with a total length of 4–9 mm; chelicerae with three promarginal and two retromarginal teeth; male palp with one patellar apophysis; RTA with pointed tip, extending beyond the distal margin of the tibia; LTA short; conductor broad and wider than tibia; median apophysis spoon-like; anterior apophysis present; epigynal teeth very long; atrium large; spermathecae simple, located posteriorly; copulatory ducts broad, located dorsal to the spermathecae.

##### Distribution.

China (Yunnan, Guangxi) (Fig. 9).

#### Flexicoelotes huyunensis

Taxon classificationAnimaliaAraneaeAgelenidae

Chen & Li sp. n.

http://zoobank.org/56FD85EE-4EA5-4740-B0ED-5BD6F4E92C26

Figs 1 , 9

##### Type material.

**Holotype** ♀: China: Yunnan Province: Wenshan Prefecture: Malipo County, Tanjiawan Village, Huyun Cave, N23°21'36", E105°02'03", elevation: 1464 m, 8.VIII.2010, Z.Y. Yao, X.X. Wang and C.X. Wu leg.

##### Etymology.

The specific name refers to the type locality; adjective.

##### Diagnosis.

**Figure 1.**
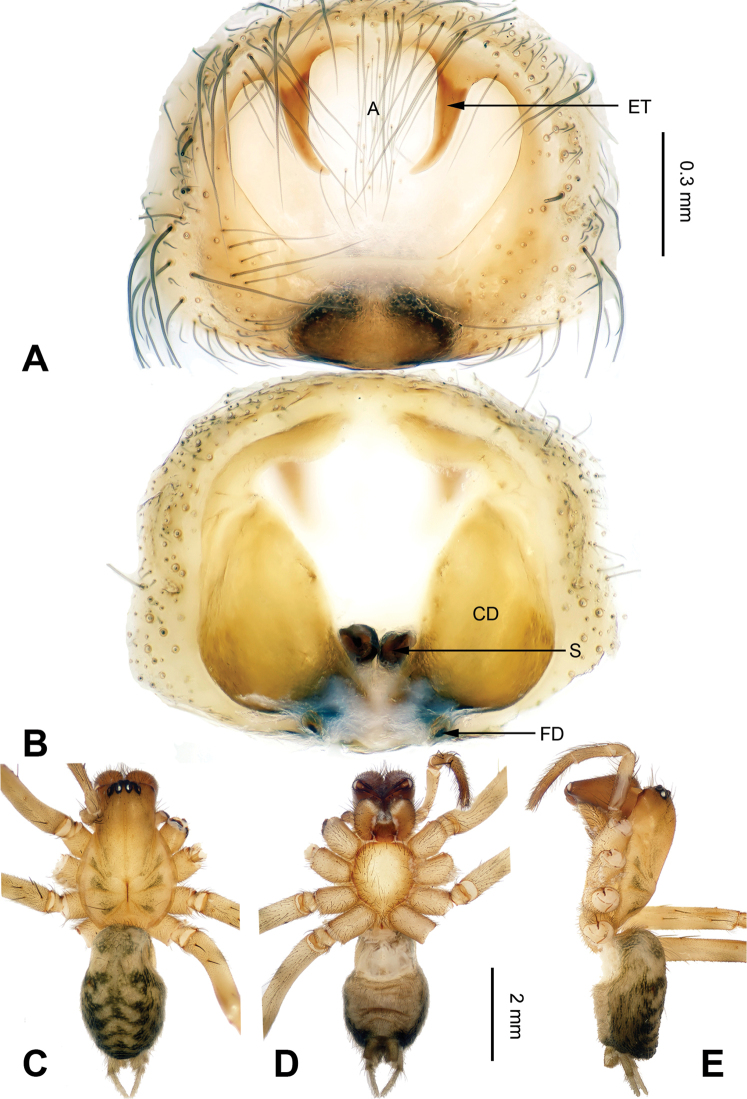
*Flexicoelotes huyunensis* sp. n., holotype female. **A** Epigyne, ventral view **B** Vulva, dorsal view **C** Female habitus, dorsal view **D** Female habitus, ventral view **E** Female habitus, lateral view. Scale bars: equal for **A**, **B**; equal for **C, D, E**.

The female can be distinguished from *Flexicoelotes jiaohanyanensis* sp. n. by the short epigynal teeth (1/2 of atrial height, whereas they are almost subequal to atrial height in related species) and the broad, short and opaque copulatory ducts (Fig. 1A–B).

##### Description.

**Female (holotype)**: Total length 6.01. Carapace 3.20 long, 2.17 wide. Abdomen 2.81 long, 1.80 wide. Eye sizes and interdistances: AME 0.09, ALE 0.17, PME 0.11, PLE 0.15; AME-AME 0.05, AME-ALE 0.03, PME-PME 0.08, PME-PLE 0.08. Leg measurements: I: 11.73 (3.09, 4.00, 2.80 1.84); II: 10.89 (2.94, 3.60, 2.66, 1.69); III: 10.04 (2.72, 3.20, 2.64, 1.48); IV: 12.90 (3.40, 4.05, 3.65, 1.80). Epigyne: atrium large, occupying 2/3 of epigynal plate; teeth long, located in atrial anterior margin, about 1/2 of atrial height and separated by their length; hoods absent; spermathecae simple, located in posterior part of epigyne; copulatory ducts broad, occupying 3/4 of epigynal plate, covering most of the spermathecae (Fig. 1A–B).

##### Distribution.

Known only from the type locality (Fig. 9).

#### Flexicoelotes jiaohanyanensis

Taxon classificationAnimaliaAraneaeAgelenidae

Chen & Li sp. n.

http://zoobank.org/707B61A7-12F8-4008-AC62-7DF270525FDF

Figs 2 , 3 , 9

##### Type material.

**Holotype** ♂: China: Guangxi Zhuang Autonomous Region: Baise City: Jingxi County, Equan Village, Jiaohanyan Cave, N23°06'22", E106°24'02", elevation: 697 m, 23.XII.2012, Z.G. Chen and Z. Zhao leg. **Paratypes**: 3♀3♂, same data as holotype.

##### Etymology.

The specific name is derived from the type locality; adjective.

##### Diagnosis.

**Figure 2.**
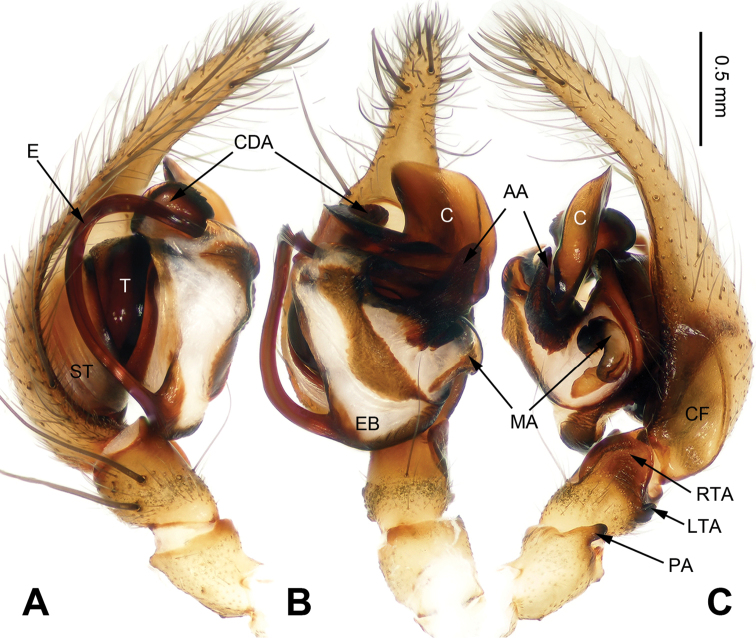
*Flexicoelotes jiaohanyanensis* sp. n., holotype male. **A** Left palp, prolateral view **B** Left palp, ventral view **C** Left palp, retrolateral view. Scale bar: equal for **A, B, C**.

**Figure 3.**
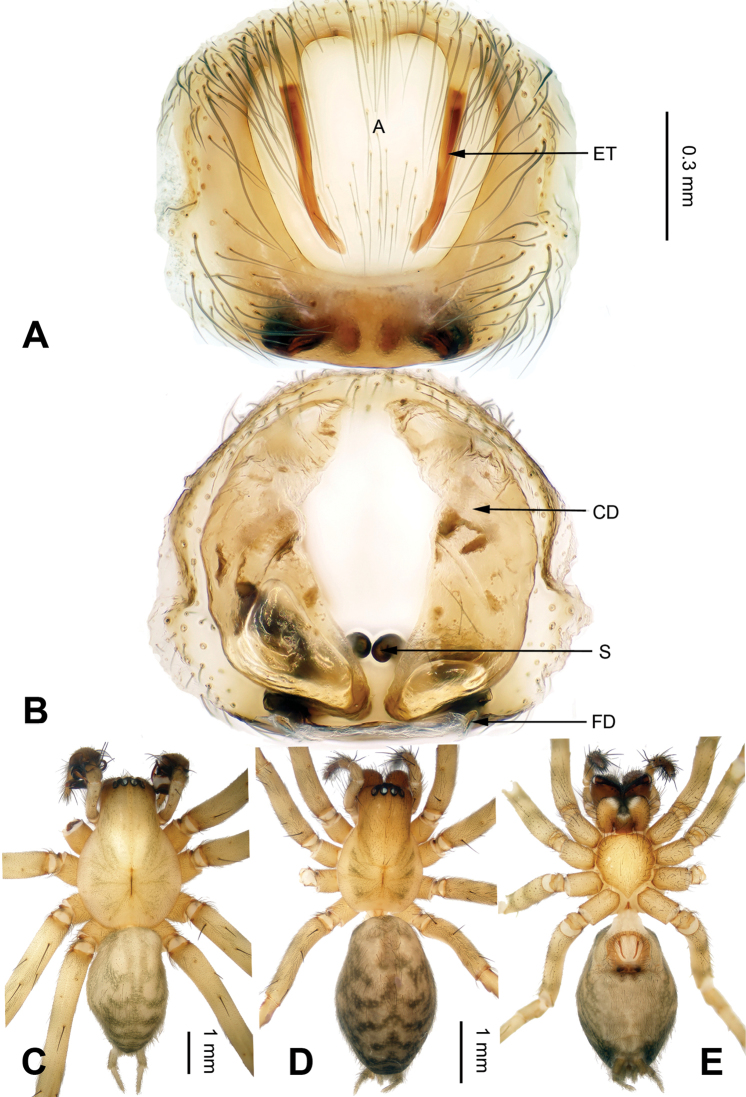
*Flexicoelotes jiaohanyanensis* sp. n., one of paratype females. **A** Epigyne, ventral view **B** Vulva, dorsal view **C** Male habitus, dorsal view **D** Female habitus, dorsal view **E** Female habitus, ventral view. Scale bars: equal for **A, B**; equal for **D, E**.

The male can be easily distinguished from other coelotines by the broad, dark conductor and the broad anterior apophysis (Fig. 2A–C). The female can be easily distinguished from other coelotines by the large atrium, occupying more than 1/2 of the epigynal plate, the very long epigynal teeth that are subequal to the height of the atrium, and the translucent copulatory ducts (Fig. 3A–B).

##### Description.

**Male (holotype)**: Total length 6.58. Carapace 3.88 long, 2.70 wide. Abdomen 3.24 long, 2.15 wide. Eye sizes and interdistances: AME 0.13, ALE 0.18, PME 0.17, P LE 0.18; AME-AME 0.04, AME-ALE 0.03, PME-PME 0.08, PME-PLE 0.07. Leg measurements: I: 15.16 (3.85, 5.10, 3.91, 2.30); II: 14.07 (3.72, 4.35, 3.76, 2.24); III: 13.13 (3.64, 3.95, 3.63, 1.91); IV: 17.05 (4.00, 5.26, 5.45, 2.34). Palp: patellar apophysis long, subequal to half of patellar width; RTA with pointed tip, extending beyond distal margin of tibia; LTA short, approximately less than 1/5 length of RTA; cymbial furrow short, about 1/4 length of cymbium; conductor broad, apex bent, with blunt tip; dorsal conductor apophysis small; median apophysis small, spoon-like; anterior apophysis broad, with blunt tip; embolus filiform, beginning at 6:30 to 7 o’clock position (Fig. 2A–C).

**Female (one of paratypes)**: Total length 4.76. Carapace 2.84 long, 1.92 wide. Abdomen 3.56 long, 2.16 wide. Eye sizes and interdistances: AME 0.11, ALE 0.18, PME 0.13, PLE 0.17; AME-AME 0.02, AME-ALE 0.03, PME-PME 0.07, PME-PLE 0.06. Leg measurements: I: 10.54 (2.88, 3.60, 2.48, 1.58); II: 9.35 (2.60, 3.12, 2.31, 1.32); III: 8.84 (2.44, 2.75, 2.43, 1.22); IV: 11.50 (3.00, 3.60, 3.36, 1.54). Epigyne: atrium large, occupying 2/3 of epigynal plate; teeth located in anterior atrial margin, separated from each other, very long, and subequal to the height of the atrium; hoods absent; spermathecae simple, located in posterior part of epigyne, covered mostly by the copulatory ducts in dorsal view; copulatory ducts broad, occupying 2/3 of epigynal plate (Fig. 3A–B).

##### Distribution.

Known only from the type locality (Fig. 9).

#### Flexicoelotes jinlongyanensis

Taxon classificationAnimaliaAraneaeAgelenidae

Chen & Li sp. n.

http://zoobank.org/ADC405C1-E049-4132-8274-5FCCC8D3930A

Figs 4 , 5 , 9

##### Type material.

**Holotype** ♂: China: Guangxi Zhuang Autonomous Region: Baise City: Napo County, Yongning Village, Jinlongyan Cave, N23°21'16", E105°51'01", elevation: 826 m, 22.XII.2012, Z.G. Chen and Z. Zhao leg. **Paratypes**: 2♀3♂, same data as holotype.

##### Etymology.

The specific name refers to the type locality; adjective.

##### Diagnosis.

**Figure 4.**
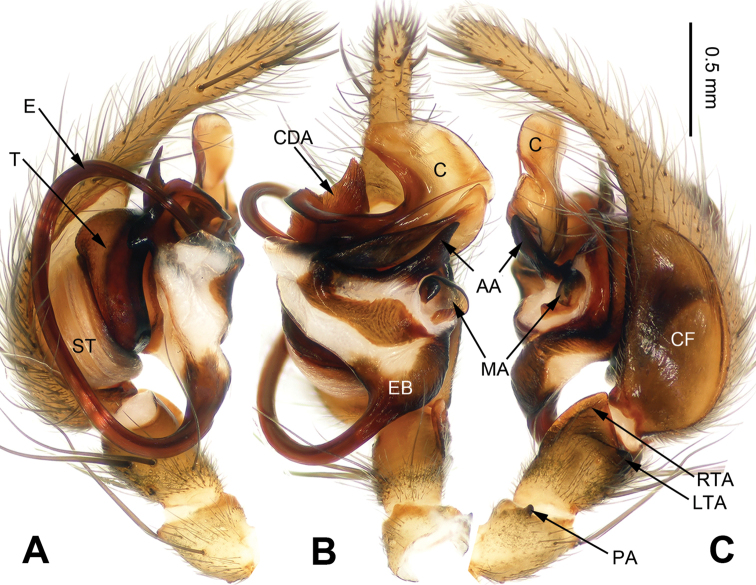
*Flexicoelotes jinlongyanensis* sp. n., holotype male. **A** Left palp, prolateral view **B** Left palp, ventral view **C** Left palp, retrolateral view. Scale bar: equal for **A, B, C**.

**Figure 5.**
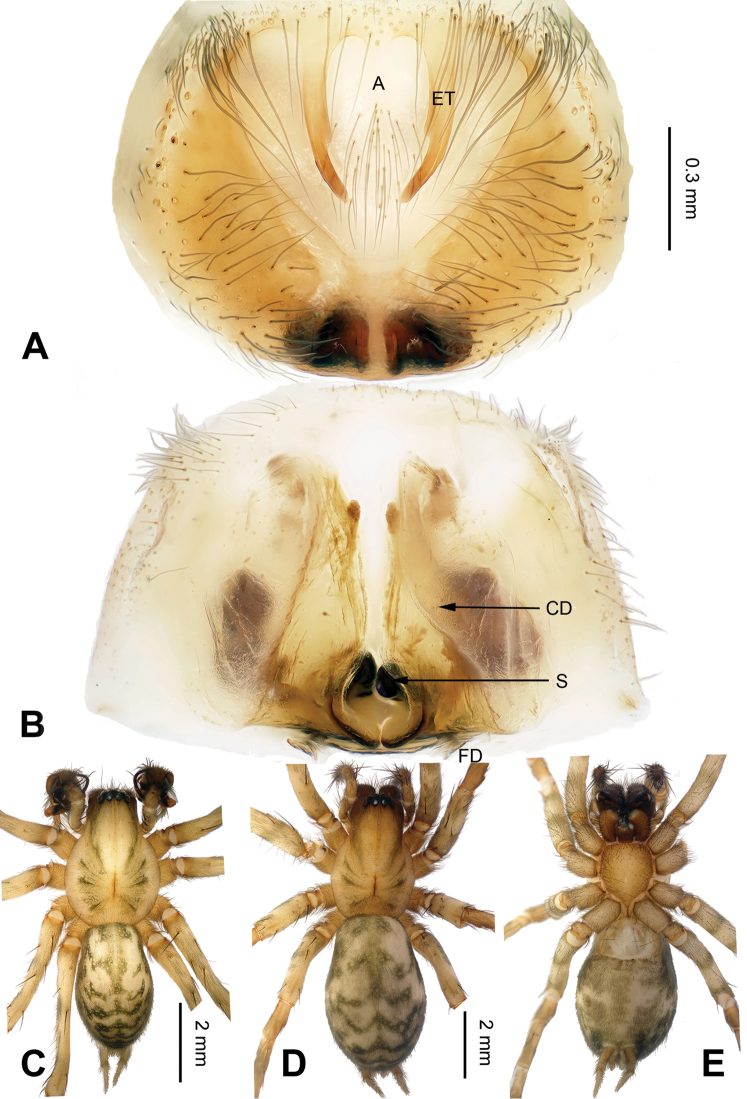
*Flexicoelotes jinlongyanensis* sp. n., one of paratype females. **A** Epigyne, ventral view **B** Vulva, dorsal view **C** Male habitus, dorsal view **D** Female habitus, dorsal view **E** Female habitus, ventral view. Scale bars: equal for **A, B**; equal for **D, E**.

The male can be distinguished from *Flexicoelotes jiaohanyanensis* sp. n. by the large dorsal conductor apophysis, the short, thin anterior apophysis, the short patellar apophysis, the complex and light-colored conductor, and the long, broad cymbial furrow (Fig. 4A–C). The female can be distinguished from *Flexicoelotes jiaohanyanensis* sp. n. and *Flexicoelotes huyunensis* sp. n. by the narrow posterior part of atrium and the unique shape of the copulatory ducts (Fig. 5A–B).

##### Description.

**Male (holotype)**: Total length 6.85. Carapace 3.55 long, 2.55 wide. Abdomen 3.30 long, 2.05 wide. Eye sizes and interdistances: AME 0.13, ALE 0.17, PME 0.17, PLE 0.19; AME-AME 0.06, AME-ALE 0.02, PME-PME 0.06, PME-PLE 0.07. Leg measurements: I: 14.35 (3.80, 4.70, 3.65, 2.20); II: 13.22 (3.52, 4.20, 3.35, 2.15); III: 12.29 (3.25, 3.68, 3.52, 1.84); IV: 16.20 (4.15, 4.85, 4.90, 2.30). Palp: patellar apophysis short; RTA with pointed tip, extending slightly beyond distal margin of tibia; LTA short, about 1/5 length of RTA; cymbial furrow long, about 1/2 length of cymbium; conductor broad, with bent apex; dorsal conductor apophysis large; median apophysis small, spoon-like; anterior apophysis short, apex is thinner than basal part; embolus filiform, beginning at 6 o’clock position (Fig. 4A–C).

**Female (one of paratypes)**: Total length 8.15. Carapace 3.55 long, 2.50 wide. Abdomen 4.60 long, 2.95 wide. Eye sizes and interdistances: AME 0.13, ALE 0.18, PME 0.18, PLE 0.17; AME-AME 0.09, AME-ALE 0.04, PME-PME 0.10, PME-PLE 0.11. Leg measurements: I: 12.44 (3.27, 4.23, 2.95, 1.99); II: 11.16 (3.08, 3.64, 2.68, 1.76); III: 10.48 (2.88, 3.28, 2.76, 1.56); IV: 13.38 (3.68, 4.25, 3.75, 1.70). Epigyne: atrium large, occupying 1/2 of epigynal plate; teeth long, located in atrial anterior margin, about 3/4 of atrial height; hoods absent; spermathecae simple, located posteriorly, covered mostly by copulatory ducts; copulatory ducts broad, occupying 3/4 of epigynal plate (Fig. 5A–B).

##### Distribution.

Known only from the type locality (Fig. 9).

#### Flexicoelotes pingzhaiensis

Taxon classificationAnimaliaAraneaeAgelenidae

Chen & Li sp. n.

http://zoobank.org/F23B1DEF-BF52-497C-B2D4-54344E36B20D

Figs 6 , 9

##### Type material.

**Holotype** ♀: China: Yunnan Province: Wenshan Prefecture: Xichou County, Pingzhai Village, Wuming Cave, N23°23'04", E104°46'28", elevation: 1405 m, 5.VIII.2010, Z.Y. Yao, X.X. Wang and C.X. Wu. leg. **Paratypes**: 2♀, same data as holotype.

##### Etymology.

The specific name refers to the type locality; adjective.

##### Diagnosis.

**Figure 6.**
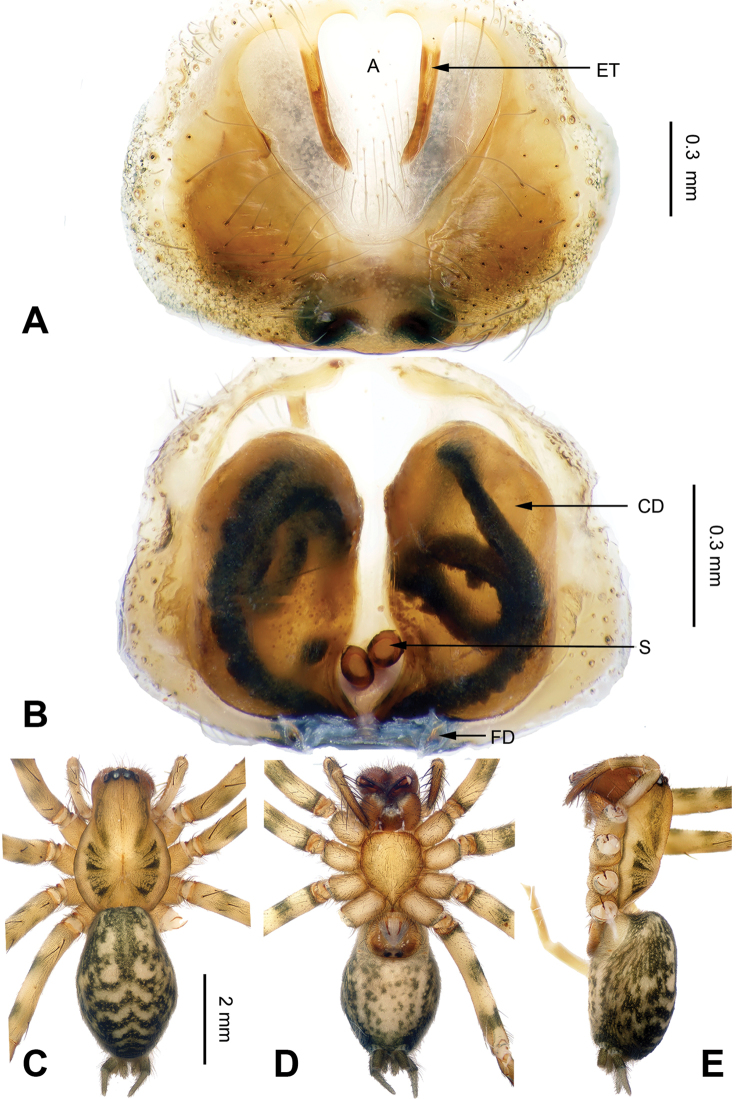
*Flexicoelotes pingzhaiensis* sp. n., holotype female. **A** Epigyne, ventral view **B** Vulva, dorsal view **C** Female habitus, dorsal view **D** Female habitus, ventral view **E** Female habitus, lateral view. Scale bars: equal for **C, D, E**.

The female can be distinguished from *Flexicoelotes jiaohanyanensis* sp. n. and *Flexicoelotes huyunensis* sp. n. by the subtriangular shape of the atrium, about 1/5 width of the anterior part, the large, oval copulatory ducts and the long, slender spermathecal stalks, and can be distinguished from *Flexicoelotes jinlongyanensis* sp. n. by the oval copulatory ducts, and the long, slender spermathecal stalks (Fig. 6A–B).

##### Description.

**Female (holotype)**: Total length 6.52. Carapace 3.16 long, 2.22 wide. Abdomen 3.36 long, 2.25 wide. Eye sizes and interdistances: AME 0.12, ALE 0.17, PME 0.16, PLE 0.17; AME-AME 0.07, AME-ALE 0.02, PME-PME 0.08, PME-PLE 0.07. Leg measurements: I: 11.16 (3.04, 3.76, 2.60 1.76); II: 10.04 (2.84, 3.20, 2.44, 1.56); III: 9.23 (2.56, 2.92, 2.40, 1.35); IV: 12.12 (3.32, 3.80, 3.40, 1.60). Epigyne: atrium large, occupying 2/3 of epigynal plate; teeth long, located in atrial anterior margin, about 2/3 of atrial height; hoods absent; spermathecae simple, located in posterior of epigyne, covered mostly by copulatory ducts; spermathecal stalks long, slender, and convoluted; copulatory ducts broad, occupying 4/5 of epigynal plate (Fig. 6A–B).

##### Distribution.

Known only from the type locality (Fig. 9).

#### Flexicoelotes xingwangensis

Taxon classificationAnimaliaAraneaeAgelenidae

Chen & Li sp. n.

http://zoobank.org/B2D49C18-8651-4491-85C9-94AEB55D879B

Figs 7 , 8 , 9

##### Type material.

**Holotype** ♂: China: Guangxi Zhuang Autonomous Region: Baise City: Debao County, Xingwang Village, Wuming Cave, N23°14'16", E106°38'35", elevation: 632 m, 19.XII.2012, Z.G. Chen and Z. Zhao. leg. **Paratypes**: 3♀2♂, same data as holotype.

##### Etymology.

The specific name refers to the type locality; adjective.

##### Diagnosis.

**Figure 7.**
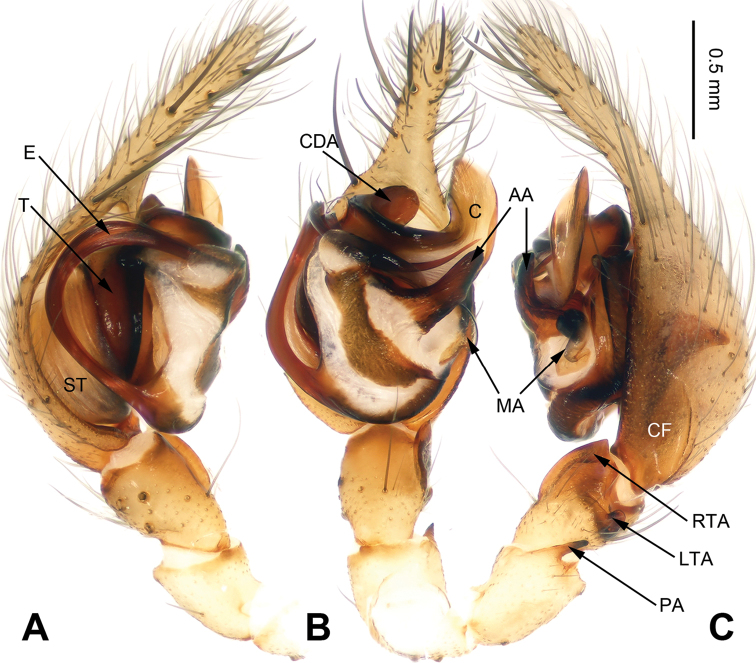
*Flexicoelotes xingwangensis* sp. n., holotype male. **A** Left palp, prolateral view **B** Left palp, ventral view **C** Left palp, retrolateral view. Scale bar: equal for **A, B, C**.

**Figure 8.**
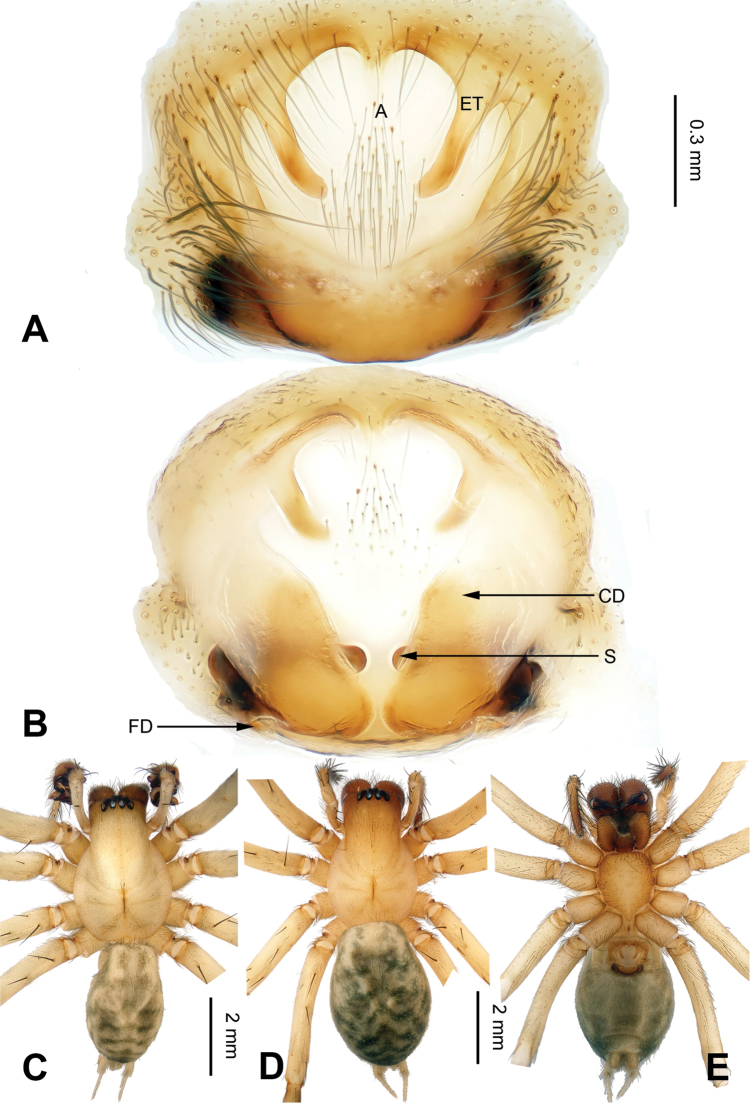
*Flexicoelotes xingwangensis* sp. n., one of paratype females. **A** Epigyne, ventral view **B** Vulva, dorsal view **C** Male habitus, dorsal view **D** Female habitus, dorsal view **E** Female habitus, ventral view. Scale bars: equal for **A, B**; equal for **D, E**.

The male can be distinguished from *Flexicoelotes jiaohanyanensis* sp. n. and *Flexicoelotes jinlongyanensis* sp. n. by the longer and more slender patellar apophysis, the thin conductor, the large, oval dorsal conductor apophysis in ventral view, and the short cymbial furrow (Fig. 7A–C). The female can be distinguished from *Flexicoelotes jiaohanyanensis* sp. n., *Flexicoelotes huyunensis* sp. n., *Flexicoelotes jinlongyanensis* sp. n., and *Flexicoelotes pingzhaiensis* sp. n. by the small and nearly hexagonal atrium, the short and light-colored epigynal teeth, and the widely separated fertilization ducts (Fig. 8A–B).

##### Description.

**Male (holotype)**: Total length 6.80. Carapace 3.72 long, 2.52 wide. Abdomen 3.08 long, 1.96 wide. Eye sizes and interdistances: AME 0.13, ALE 0.20, PME 0.15, PLE 0.17; AME-AME 0.05, AME-ALE 0.02, PME-PME 0.06, PME-PLE 0.09. Leg measurements: I: 16.01 (4.29, 5.19, 3.97, 2.56); II: 14.56 (3.85, 4.68, 3.78, 2.25); III: 13.50 (3.60, 4.05, 3.90, 1.95); IV: 17.57 (4.50, 5.26, 5.45, 2.36). Palp: patellar apophysis long, subequal to patellar width; RTA with pointed tip, extending beyond distal margin of tibia; LTA long, about 1/3 length of RTA; cymbial furrow short, about 1/5 length of cymbium; conductor broad, with bent apex; dorsal conductor apophysis large; median apophysis small, spoon-like; anterior apophysis broad, with blunt tip; embolus filiform, beginning at 7:30 o’clock position (Fig. 7A–C).

**Female (one of paratypes)**: Total length 7.64. Carapace 3.64 long, 2.53 wide. Abdomen 4.00 long, 2.78 wide. Eye sizes and interdistances: AME 0.13, ALE 0.20, PME 0.15, anterior apophysis PLE 0.17; AME-AME 0.06, AME-ALE 0.04, PME-PME 0.10, PME-PLE 0.11. Leg measurements: I: 13.48 (3.65, 4.60, 3.25, 1.98); II: 12.31 (3.40, 4.05, 3.05, 1.81); III: 11.40 (3.24, 3.60, 3.08, 1.48); IV: 15.46 (4.05, 4.75, 4.88, 1.78). Epigyne: atrium large, occupying 2/3 of epigynal plate; teeth long, located in atrial anterior margin, about 2/3 of atrial height; hoods absent; spermathecae simple, located posteriorly; copulatory ducts broad, occupying 2/3 of epigynal plate, covering most of spermathecae (Fig. 8A–B).

##### Distribution.

**Figure 9.**
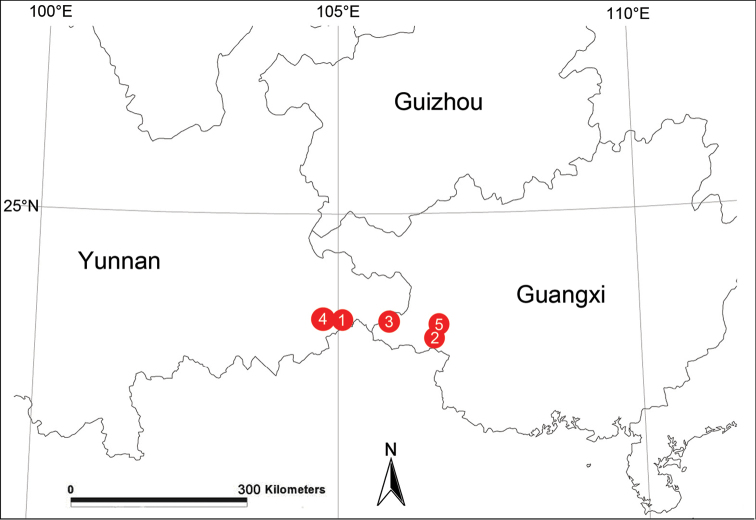
Localities of new *Flexicoelotes* species from China. **1** *Flexicoelotes huyunensis* sp. n. **2** *Flexicoelotes jiaohanyanensis* sp. n. **3** *Flexicoelotes jinlongyanensis* sp. n. **4** *Flexicoelotes pingzhaiensis* sp. n. **5** *Flexicoelotes xingwangensis* sp. n.

Known only from the type locality (Fig. 9).

## Supplementary Material

XML Treatment for Flexicoelotes

XML Treatment for Flexicoelotes huyunensis

XML Treatment for Flexicoelotes jiaohanyanensis

XML Treatment for Flexicoelotes jinlongyanensis

XML Treatment for Flexicoelotes pingzhaiensis

XML Treatment for Flexicoelotes xingwangensis
